# Application progress of ensemble forecast technology in influenza forecast based on infectious disease model

**DOI:** 10.3389/fpubh.2023.1335499

**Published:** 2023-12-15

**Authors:** Lianglyu Chen

**Affiliations:** Chongqing Institute of Meteorological Sciences, Chongqing, China

**Keywords:** influenza, ensemble forecast, infectious disease, numerical weather forecast, respiratory disease

## Abstract

To comprehensively understand the application progress of ensemble forecast technology in influenza forecast based on infectious disease model, so as to provide scientific references for further research. In this study, two keywords of “influenza” and “ensemble forecast” are selected to search and select the relevant literatures, which are then outlined and summarized. It is found that: In recent years, some studies about ensemble forecast technology for influenza have been reported in the literature, and some well-performed influenza ensemble forecast systems have already been operationally implemented and provide references for scientific prevention and control. In general, ensemble forecast can well represent various uncertainties in forecasting influenza cases based on infectious disease models, and can achieve more accurate forecasts and more valuable information than single deterministic forecast. However, there are still some shortcomings in the current studies, it is suggested that scientists engaged in influenza forecast based on infectious disease models strengthen cooperation with scholars in the field of numerical weather forecast, which is expected to further improve the skills and application level of ensemble forecast for influenza.

## 1 Introduction

Influenza is a respiratory disease caused by influenza virus infection, it’s highly contagious and its outbreaks have the characteristics of seasonal circulation. According to statistics, worldwide, influenza epidemics cause about 3–5 million severe cases of lower respiratory tract infection and 250,000–690,000 deaths every year (1), which poses a great threat to human public health. During the influenza epidemics, a large number of patients not only cause a serious burden on the medical resources, but also cause huge social and economic burdens.

Accurately forecasting the occurrence and development of influenza has important scientific significance for governments to formulate specific vaccination and non-drug interventions, prepare adequate medical resources in advance, and evaluate the effect of policies. Forecasting influenza cases based on infectious disease model is an important method for scientific prevention and control. Taking the widely used susceptible–infectious–recovered–susceptible (SIRS) model as an example (2), infectious disease model is usually composed of ordinary differential equations that characterize the dynamic mechanism of infectious disease transmission, and contains some sensitive parameters, such as infection rate (i.e., the probability of a patient to infect others), the probability of conversion from a latent period person to an infected person, the recovery rate of infected persons, the mortality rate and the coefficient of government interventions. After setting the relevant sensitivity parameters and the initial values of the differential equations (such as the number of cases at present, etc.) in advance, the number of influenza cases in the future can be achieved by numerical integration of the differential equations.

After decades of continuous development, the infectious disease models have shown good potentials for application. However, the initial values in infectious disease models still inevitably have certain errors, and the relevant sensitivity parameters in the models are all set according to users’ experiences. Due to the high nonlinearity of infectious disease models, the error of the initial values and the relevant sensitive parameters will be amplified with the extension of forecast lead time and eventually lead to large biases of the forecast results, which limits the accuracy of the model forecast results to a certain extent. Therefore, it is worthy to quantitatively reflect the uncertainty of the initial values and sensitive parameters in infectious disease models, thus to solve the uncertainty problems in the single deterministic forecast result and improve the accuracy and application level of the infectious disease model forecasts. In view of this, learning from and applying the ensemble forecast technology developed in the field of numerical weather forecast is expected to effectively solve the above problems.

In recent years, it is noticed that the ensemble forecast technology has been applied in forecasting influenza cases based on infectious disease models, this paper will review the literature. Two keywords of “influenza” and “ensemble forecast” are selected to search and select the relevant literatures, which are then outlined and summarized. In addition, some suggestions are put forward, according to the author’s experiences in research and application of ensemble forecast technology for several years.

## 2 Introduction of ensemble forecast technology

**Figure 1:**
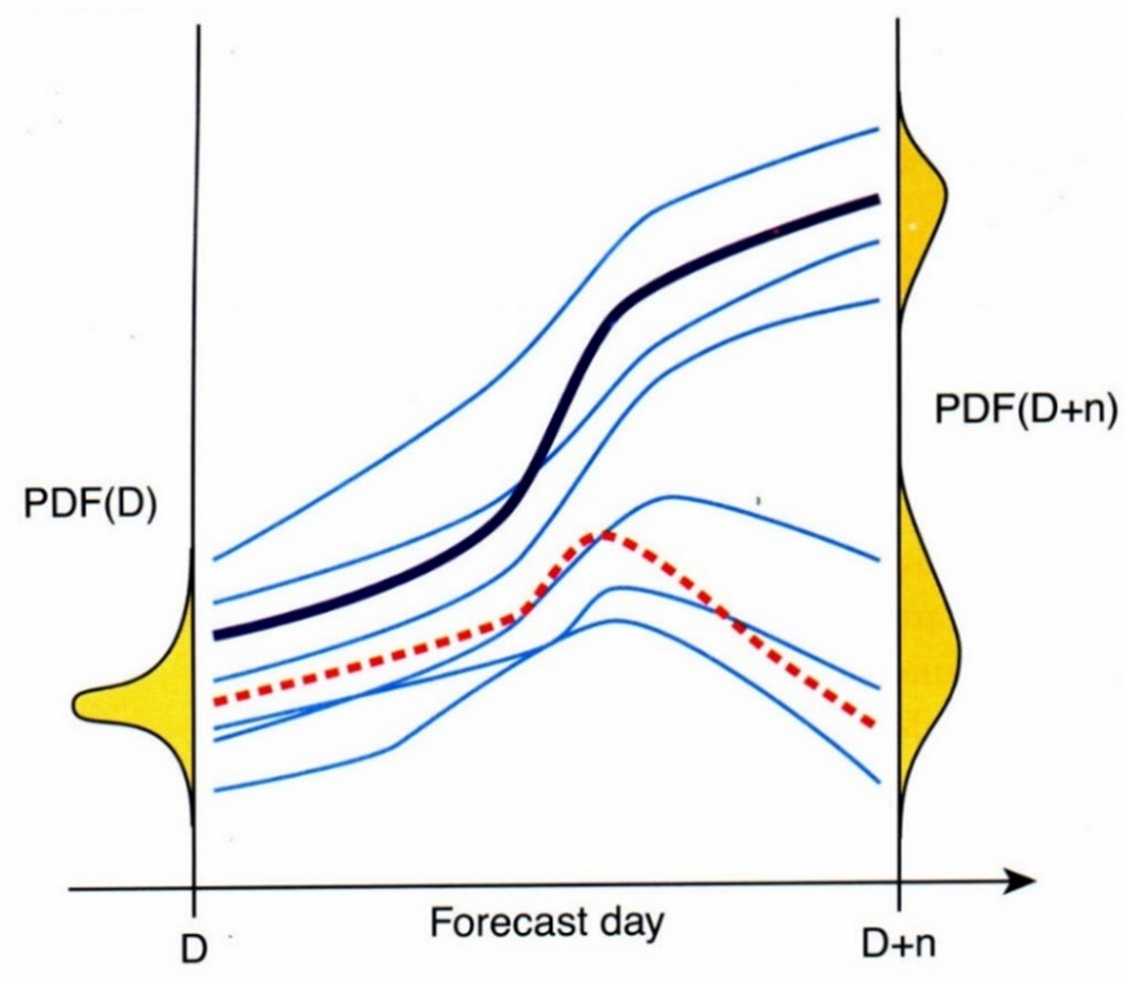
The schematic diagram of ensemble forecast (Black line: deterministic forecast; Blue line: ensemble members’ forecasts; Red dotted line: observation).

Ensemble forecast technology is developed in the field of numerical weather forecast. The essence of numerical weather forecast is to calculate the forecast value in the future by repeatedly integrating the differential equations representing the atmospheric motion started from the initial values, which is consistent with the essence of forecasting influenza cases based on infectious disease models. Due to the chaotic characteristics of the atmosphere, any small error in the initial values may quickly diverge the outcomes after a period of integration, and sometimes may even result in completely opposite results. In order to solve the above problems, the concept of ensemble forecast was put forward in the 1970s (3): Based on a certain mathematical method, a set of initial values with certain probability density function (PDF) distribution characteristics are firstly generated (as shown in Figure 1), each initial value may represent the real condition of the atmosphere. After this, ensemble forecast results can be achieved by numerical integration of each initial value (usually combined with different physical process parameterization schemes, planetary boundary layer conditions or even based on different models), thus to inferring the evolution of the PDF of atmospheric states over different forecast lead time.

Ensemble forecast is no longer single deterministic forecast, but a group of forecasts, each of which can be called an ensemble member, and the divergence degree of ensemble members’ forecasts (i.e., the ensemble spread) can be used as a quantitative representation of the forecast uncertainty (i.e., the forecast error). Appropriate post processes for ensemble members’ forecasts can achieve corresponding post-processed deterministic forecast products, and the forecast performance of these products are usually significantly better than that of the original single deterministic forecast. In addition, modern ensemble forecasts are expressed probabilistically other than deterministically, more decision mistakes could be avoided if the decisions are made based on whether the probabilities exceed some prior determined threshold for action, which is an important aspect for the application of ensemble forecast technology.

Ensemble forecast has become a relatively mature technology in the field of numerical weather forecast, and has been widely used in the operational forecasting practice (4). Meanwhile, as a scientific way to solve the uncertainty problems existing in single deterministic forecast, it has also been widely used in the fields of aviation (5), biology (6), hydrology (7), electricity (8), economy (9) and infectious disease prevention and control in recent years, providing great enlightening significance for solving the prediction problems in related fields.

## 3 Application progress of influenza ensemble forecast

### 3.1 Application progress of influenza ensemble forecast in the United States

The United States is one of the country’s most seriously affected by seasonal influenza, and the Department of Environmental Health Sciences of Columbia University has carried out several studies on influenza ensemble forecast for some megacities in the past decade.

Shaman and Karspeck (2) established an influenza ensemble forecast system based on the SIRS model and ensemble adjusted Kalman filter (EAKF) assimilation technology developed in the field of numerical weather forecast. This system uses EAKF assimilation method to assimilate the data of current influenza cases updated on relevant websites in real time, thus to generate 250 sets of initial values, the SIRS model is then used to integrate the initial values to achieve 250 sets of forecast values. On this basis, the ensemble forecast system was tested and evaluated for forecasting influenza cases in New York City from 2003 to 2008, In general, the influenza ensemble forecast system can accurately forecast the peak timing about 7 weeks in advance of the actual peak, and the spread of the ensemble members’ forecasts can be used to enhance the confidence in the accuracy of forecast results.

In the influenza epidemic seasons of 2012 and 2013, the above-mentioned influenza ensemble forecast system (2) was operationally implemented in real time and provided forecast results of influenza cases in 108 cities of the United States (10), which was the first operational ensemble forecast system for influenza. According to the related evaluation results: The influenza ensemble forecast system could accurately forecast the peak timing about 9 weeks in advance of the actual peak. In general, the forecast accuracy gradually increased with the season progressed. By the 52th week, prior to peak for the majority of cities, 63% of all ensemble forecasts were accurate.

The nonlinear growth of errors is the main source of forecast errors in infectious disease models. In order to further optimize the influenza ensemble forecast system, on the basis of the previous works, Pei and Shaman (11) quantitatively estimated the nonlinear error results of the above-mentioned influenza ensemble forecast system through the error breeding analysis method and then accordingly corrected the forecast errors. After this, the ensemble forecast experiments for influenza cases in 95 cities of the United States from 2003 to 2008 were conducted, evaluation results indicate that: In general, through the nonlinear error correction process, the forecast accuracy of the peak time and peak intensity of influenza outbreak are both improved.

On the basis of the previous works, Pei et al. (12) found that the initial value error and random error in the infectious disease model have similar growth characteristics in the process of model integration through several diagnostic analysis processes, which further confirmed that the nonlinear dynamic error growth is the main source of the forecast error of infectious disease models. On this basis, the direction of the fastest growth of initial value error was found by singular vector analysis method and then accordingly used to optimize the initial value perturbation scheme. After this, the ensemble spread increases significantly so that the forecast uncertainty could be better represented, and the ensemble forecast accuracy is also further improved.

To sum up, the United States is the country with the most research on influenza ensemble forecast technology. In recent years, an influenza ensemble forecast system was built, and some ensemble forecast researches such as forecast results evaluation, error evolution characteristic diagnosis and analysis, ensemble forecasting initial value perturbation scheme optimization have been done. The newly-developed influenza ensemble forecast system has been operationally implemented and provided reference for scientific prevention and control.

### 3.2 Application progress of influenza ensemble forecast in subtropical regions

Influenza outbreaks in temperate regions usually present the characteristics of seasonal circulation, while that in tropical and subtropical regions presents irregular non-seasonal distribution characteristics and can breakout throughout the year. Therefore, the forecast of influenza cases in tropical and subtropical regions is more difficult.

Yang et al. (13) established an influenza ensemble forecast system with ensemble size of 500 for the Hong Kong city in subtropical region based on the SIRS model and the EAKF assimilation technology, which is similar to the ensemble forecast system constructed by Shaman and Karspeck (2). Based on this, the ensemble forecast system was tested and evaluated for influenza cases in Hong Kong from 1998 to 2013. Overall, the influenza ensemble forecast system was able to predict the peak timing and peak intensity of 44 influenza pandemics caused by single influenza strain or multiple influenza strains in the past 16 years. The overall forecast accuracy of 1–3 weeks in advance was 37%, and the forecast accuracy increased with the ensemble spread. The maximum accuracy of the peak time (intensity) of the pandemic caused by different strains is 43–93% (45–89%). In general, for non-seasonal influenza pandemics in subtropical regions, which are difficult to predict, the influenza ensemble forecast system can forecast accurately at least three weeks in advance.

The influenza ensemble forecast system for Hong Kong is generally similar to that established by the Department of Environmental Health Sciences of Columbia University, but its overall forecast accuracy is obviously worse, which may be mainly due to the lower predictability of influenza outbreaks in subtropical regions compared to temperate regions.

### 3.3 Application progress of super ensemble forecast technology for influenza

In addition to establishing ensemble forecast system based on a single model, the forecast results based on different models can be directly combined to form ensemble forecasts, which is called multi-model super ensemble forecast in the field of numerical weather forecast. Generally speaking, each model has its certain advantages and disadvantages. Thus, the super ensemble forecast may absorb (avoid) the advantages (disadvantages) of each single model, so as to achieve more accurate forecast results. In recent years, several studies have been fulfilled on the multi-model super ensemble forecast for influenza.

To incorporate all available data and methods to achieve a more accurate forecast of influenza cases, the Centers for Disease Control and Prevention of the United States has organized seasonal influenza forecasting challenges since the 2013 season. In the 2017 and 2018 influenza seasons, the 22 teams participating in the challenge combined the forecast results of their respective model through the machine learning method (14), and the specific weights for each model were determined by its forecast accuracy in previous seasons. It is found that the forecast results after weighted integration are obviously better than that of the 22 teams, which shows good potentials to be operationally implemented.

Yamana et al. (15) also completed a similar study on the seasonal influenza, but during the weighted integration process based on the multi-model super ensemble forecast results, the same weight was applied to each model. The results showed that the forecast results of the multi-model ensemble forecasts outperform those of each single model, and very poor forecast results were less likely to occur.

Different from the above schemes for determining weight of each single model, McAndrew and Reich (16) generated the weights of each model by its forecast accuracy updated weekly in real time and found that the forecast accuracy based on this weighting scheme are better than that of the above-mentioned two schemes (14, 15).

To sum up, scheme for determining weight should be selected according to specific needs or situations when carrying out weighted integration processes for multi-model super ensemble forecast results, since each scheme has its own advantages and disadvantages. In general, the development of super ensemble forecast and proper weighted integration process could achieve more accurate forecast results.

## 4 Discussion

In recent years, several influenza ensemble forecast systems were established and some related researches were conducted such as forecast results evaluation, error evolution characteristic diagnosis and analysis, ensemble forecasting initial value perturbation scheme optimization, super ensemble forecast and so on. Some well-performed influenza ensemble forecast systems have been operationally implemented and provided references for scientific prevention and control. In general, ensemble forecast can represent various uncertainties in forecasting influenza cases based on infectious disease model and achieve more accurate forecasts and more valuable information than the single deterministic forecast, showing a good prospect for application. In addition, the development of super ensemble forecast and proper weighted integration process could achieve more accurate forecast results.

However, there are still some weakness in the above-mentioned works: Firstly, some of the above-mentioned influenza ensemble forecast systems use the EAKF assimilation method to generate initial values. In fact, there are many other initial value perturbation technologies (17) in the field of numerical weather forecast that can be applied to establish influenza ensemble forecast system, which are expected to reflect the forecast uncertainty of infectious disease model more reasonably and improve the corresponding ensemble forecast skills; Secondly, at present, the post process technologies for influenza ensemble forecast products are mostly simple ensemble average or weighted average based on super ensemble forecast. It is expected to further improve the accuracy and application level of influenza ensemble forecast products by learning to and applying other mature post-process technologies (18) in the field of numerical weather forecast, such as the probability-matching ensemble mean, merged optimal ensemble quantile and Bayesian average; Thirdly, modern ensemble forecasts are expressed probabilistically other than deterministically, more decision mistakes could be avoided if the decisions are made based on whether the probabilities exceed some prior determined threshold for action, which is an important aspect for the application of ensemble forecast technology (19). However, at present, probability forecast is rarely used in the influenza ensemble forecast system, strengthening the application of ensemble probability forecast is expected to further improve the application level of influenza ensemble forecast and reduce decision-making errors.

To further improve the skills and application level of ensemble forecast for influenza, I strongly suggest that scientists engaged in influenza forecast based on infectious disease models should strengthen cooperation with scientists in the field of numerical weather forecast, which is expected to produce innovative academic ideas and achieve new breakthroughs through interdisciplinary cooperation.

Due to the limitation of words, this study only reviews the application progress of ensemble forecast technology in influenza forecast based on infectious disease model. In fact, there are many other similar studies involving other infectious diseases such as dengue (20) and COVID-19 (21), which may be reviewed in the future.

## Author contributions

LC: Writing – original draft, Writing – review & editing.

## References

[1] Iuliano AD Roguski KM Chang HH Muscatello DJ Palekar R Tempia S. Estimates of global seasonal influenza-associated respiratory mortality: a modelling study. Lancet. (2018) 391:1285–00. doi: 10.1016/S0140-6736(17)33293-2, PMID: 29248255 PMC5935243

[2] Shaman J Karspeck A. Forecasting seasonal outbreaks of influenza. Proc Natl Acad Sci USA. (2012) 109:20425–30. doi: 10.1073/pnas.1208772109, PMID: 23184969 PMC3528592

[3] Leith CE. Theoretical skill of Monte Carlo forecasts. Mon Wea Rev. (1974) 102:409–18. doi: 10.1175/1520-0493(1974)102<0409:TSOMCF>2.0.CO;2

[4] Lewis JM. Roots of ensemble forecasting. Mon. Wea. Rev. (2005) 133:1865–85. doi: 10.1175/MWR2949.1

[5] Storer LN Gill PG Williams PD. Multi-model ensemble predictions of aviation turbulence. Meteorol Appl. (2018) 26:416–28. doi: 10.1002/met.1772

[6] Shawn MC Solomon ZD Alison RM. Evaluating ensemble forecasts of plant species distributions under climate change. Ecol Model. (2013) 266:126–30. doi: 10.1016/j.ecolmodel.2013.07.006

[7] Li WT Duan QY Miao CY Ye A Gong W di Z. A review on statistical postprocessing methods for hydrometeorological ensemble forecasting. Wires Water. (2017) 4:e1246. doi: 10.1002/wat2.1246

[8] Thorey J Chaussin C Mallet V. Ensemble forecast of photovoltaic power with online CRPS learning. Int J Forecast. (2018) 34:762–73. doi: 10.1016/j.ijforecast.2018.05.007

[9] João AB. Ensemble predictions of recovery rates. J Financ Serv Re. (2014) 46:177–93. doi: 10.1007/s10693-013-0165-3

[10] Shaman J Karspeck A Yang W Tamerius J Lipsitch M. Real-time influenza forecasts during the 2012–2013 season. Nat Commun. (2013) 4:2837. doi: 10.1038/ncomms3837, PMID: 24302074 PMC3873365

[11] Pei S Shaman J. Counteracting structural errors in ensemble forecast of influenza outbreaks. Nat Commun. (2017) 8:925. doi: 10.1038/s41467-017-01033-1, PMID: 29030543 PMC5640637

[12] Pei S Cane MA Shaman J. Predictability in process-based ensemble forecast of influenza. PLoS Comput Biol. (2019) 15:e1006783. doi: 10.1371/journal.pcbi.1006783, PMID: 30817754 PMC6394909

[13] Yang W Cowling BJ Lau EHY Shaman J. Forecasting influenza epidemics in Hong Kong. PLoS Comput Biol. (2015) 11:e1004383. doi: 10.1371/journal.pcbi.1004383, PMID: 26226185 PMC4520691

[14] Reich NG McGowan CJ Yamana TK Tushar A Ray EL Osthus D. Accuracy of real-time multi-model ensemble forecasts for seasonal influenza in the U.S. PLoS Comput Biol. (2019) 15:e1007486. doi: 10.1371/journal.pcbi.1007486, PMID: 31756193 PMC6897420

[15] Yamana TK Kandula S Shaman J. Individual versus superensemble forecasts of seasonal influenza outbreaks in the United States. Public Libr Sci Comp Biol. (2017) 13:e1005801. doi: 10.1371/journal.pcbi.1005801, PMID: 29107987 PMC5690687

[16] McAndrew T Reich NG. Adaptively stacking ensembles for influenza forecasting. Stat Med. (2021) 40:6931–52. doi: 10.1002/sim.9219, PMID: 34647627 PMC8671371

[17] Wang X Bishop C. A comparison of breeding and ensemble transform Kalman filter ensemble forecast schemes. J Atmos Sci. (2003) 60:1140–58. doi: 10.1175/1520-0469(2003)060<1140:ACOBAE>2.0.CO;2

[18] Qiao X Wang S Schwartz CS Liu Z Min J. A method for probability matching based on the ensemble maximum for quantitative precipitation forecasts. Mon. Wea. Rev. (2020) 148:3379–96. doi: 10.1175/MWR-D-20-0003.1

[19] Joslyn S Pak K Jones D Pyles J Hunt E. The effect of probabilistic information on threshold forecasts. Weather Forecast. (2007) 22:804–12. doi: 10.1175/WAF1020.1

[20] Buczak AL Baugher B Moniz LJ Bagley T Babin SM Guven E. Ensemble method for dengue prediction. PLoS One. (2018) 13:e0189988. doi: 10.1371/journal.pone.0189988, PMID: 29298320 PMC5752022

[21] Cramer EY Ray EL Lopez VK Bracher J Brennen A Castro Rivadeneira AJ. Evaluation of individual and ensemble probabilistic forecasts of COVID-19 mortality in the United States. Proc Natl Acad Sci USA. (2022) 119:e2113561119. doi: 10.1073/pnas.2113561119, PMID: 35394862 PMC9169655

